# Bone Remodeling in Osteoarthritis—Biological and Radiological Aspects

**DOI:** 10.3390/medicina59091613

**Published:** 2023-09-07

**Authors:** Luka Dudaric, Ivo Dumic-Cule, Eugen Divjak, Tomislav Cengic, Boris Brkljacic, Gordana Ivanac

**Affiliations:** 1 Croatia Poliklinika, Rijeka Radiology Unit, Vukovarska 7A, 51000 Rijeka, Croatia; lukadudaric@yahoo.com; 2 Clinical Department of Diagnostic and Interventional Radiology, University Hospital Centre Zagreb, Kispaticeva 12, 10000 Zagreb, Croatia; ivodc1@gmail.com; 3 Department of Nursing, University North, 104 Brigade 3, 42000 Varazdin, Croatia; 4 Department of Diagnostic and Interventional Radiology, University Hospital Dubrava, Avenija Gojka Suska 6, 10000 Zagreb, Croatia; edivjak@gmail.com (E.D.); boris@brkljacic.com (B.B.); gordana.augustan@gmail.com (G.I.); 5 Department of Orthopedics and Traumatology, University Hospital Centre Sestre Milosrdnice, Draskoviceva 19, 10000 Zagreb, Croatia; 6 School of Medicine, University of Zagreb, Salata 3, 10000 Zagreb, Croatia

**Keywords:** osteoarthritis, osteophyte, bone remodeling

## Abstract

Among available papers published on the given subject over the last century, various terms have been used as synonyms for one, now generally accepted—osteoarthritis, in some countries called “wear and tear” or “overload arthritis”. The opsolent terms—hypertrophic arthritis, degenerative arthritis, arthritis deformans and osteoarthrosis—sought to highlight the dominant clinical signs of this ubiquitous, polymorph disease of the whole osteochondral unit, which by incidence and prevalence represents one of the leading chronic conditions that cause long-term pain and incapacity for work. Numerous in vitro and in vivo research resulted in broadened acknowledgments about osteoarthritis pathophysiology and pathology on both histological and cellular levels. However, the cause of osteoarthritis is still unknown and is currently the subject of a hypothesis. In this paper, we provide a review of recent findings on biological phenomena taking place in bone tissue during osteoarthritis to the extent useful for clinical practice. Choosing a proper radiological approach is a conditio sine qua non to the early diagnosis of this entity.

## 1. Introduction

Bone morphogenesis (osteogenesis) is the process of formation and maintenance of bone tissue and is the result of bone formation and bone resorption. Both processes, bone formation (osteoproduction) and bone resorption (osteoresorption), are functionally balanced in the creation and maintenance of optimal functional structure, or homeostasis, of the skeletal system according to functional demands. Deviation from the physiological balance of these processes is manifested in pathological osteogenesis. One morphological substrate of pathological osteogenesis is osteophyte formation in osteoarthritis (OA).

In other words, physiological and pathological osteogenesis are essentially similar processes as they are based on the same basic principles of bone tissue biology—osteoinduction and osteoconduction. The principle of osteoinduction is based on molecular factors that act on the proliferation and differentiation of bone phenotype cells [1,2,3,4]. The purpose of lifelong continuous internal reconstruction of bone tissue and the skeletal system is to achieve and maintain optimal skeletal architecture according to mechanical, static, and humoral circumstances. During prenatal, neonatal, and infantile stages, bones undergo development and growth. Intensive development of all systems of the musculoskeletal system occurs in early childhood and school age, which ends around 14 years of age. The maturation period, from 15 to 20 years of age, is characterized by the overall growth of the body, with bones rapidly growing in length and reaching their anatomical norm, definitive shape, and size through bone modeling. At the end of this period and entering adulthood, the processes of organ development conclude, and bone growth ceases. The intensity of bone production decreases and balances with bone resorption, and the total mass of bone tissue stabilizes and undergoes permanent lifelong remodeling. During aging, bone resorption intensifies, bones atrophy, lose strength and elasticity, and degenerative changes and senile osteoporosis occur.

Histomorphological osteogenic changes occur through processes that are always identical, regardless of the circumstances or phases of growth and development and the modeling or remodeling of bones. During the embryonic period, bones develop from embryonic connective tissue. The organization and morphology of the skeleton in development are determined by a series of programmed and induced processes. The process of osteogenesis or ossification occurs in two ways: intramembranous and endochondral ossification. Ossification also occurs postnatally [1,2,3,4].

In mature human individuals, approximately 25% of cancellous bone tissue and about 3% of compact bone tissue are replaced by remodeling each year [5]. The purpose of bone remodeling is to optimize the architecture of the skeletal system and adapt it to biomechanical demands. It is a dynamic lifelong process that achieves and maintains homeostasis of the skeletal system according to changing biomechanical and metabolic circumstances. Haapasalo et al. demonstrated the great capacity for modifying bone architecture in their study of changes in size, shape, and distribution of bone mass in the humerus of professional tennis players [6]. Significant differences were found between the right and left arms of the subjects. The dominant arm of tennis players had increased bone strength due to increased bone size, while bone volume density did not contribute to this difference compared to the non-dominant arm. In other words, the biomechanical optimization of the dominant arm’s humerus was not the result of increased bone mass but rather specific architecture achieved through bone mass remodeling in accordance with the biomechanical demands of playing tennis. Similar conclusions were drawn by Bass et al. in their study of biomechanical loading of the humerus in pre- and post-pubertal girls playing tennis [7]. Biomechanical loading before puberty led to increased bone mass and resistance to bending. The increased resistance to bending and torsion was primarily achieved by changing the shape and mass of individual bone parts, rather than changing the volumetric bone density or its total mass. The dominant humerus adapted to the increased load through increased bone resorption in the endocortical area.

Bone remodeling achieves optimal biomechanical adaptation of bone with a minimal amount of bone material. If bone strength was achieved solely by increasing bone mass, an excessively massive skeletal system would burden the organism in all aspects of its biological functionality (energy and substance expenditure, mobility, etc.). In their study on the shape of the femoral neck in women, Zebaze et al. showed that its fragility in older age is not solely due to bone loss but also inadequate remodeling of existing bone mass [8].

## 2. Bone Modeling and Remodeling in Osteoarthritis

Two layers can be distinguished in the subchondral bone tissue histomorphologically. The layer of bone tissue (thickness 1–3 mm) that continues from the calcified layer of articular cartilage is composed of homogeneous compact bone material. Mechanically and physiologically, this layer corresponds to compact bone tissue in other parts of the bone [8,9,10,11,12]. This layer is followed by a more porous and metabolically active layer of trabecular bone tissue, which has lower density and volume. When studying osteoarthritic changes in the subchondral bone tissue, both layers should be distinguished due to their different biological responses in later stages of osteoarthritis [13,14,15]. Morphological changes during the progression of osteoarthritis also affect the articular cartilage. Changes in the zone of calcified cartilage differ from those affecting its more superficial zones separated by the tidemark.

In the early stages of osteoarthritis, there is a significant increase in remodeling of the subchondral bone tissue. The mineral apposition rate exceeds 3.5 μm/day, which is about five times higher compared to physiological remodeling. The consequences of increased remodeling during osteoarthritis include a temporary reduction in bone mass, increased porosity, and decreased bone density. Additionally, the number of areas in the subchondral bone that undergo remodeling increases [16]. In an experiment on mice, Zhu et al. demonstrated that osteoclasts in the affected subchondral bone tissue produce netrin-1, which contributes to pain induction by stimulating sensory nerve fibers in the subchondral bone tissue [17].

Significant thinning of the subchondral bone tissue layer has been observed in an experimental model of osteoarthritis in dogs. This finding was accompanied by significant destruction of the articular cartilage and reduced production of glycosaminoglycans within it [18]. Similar changes were reported by Bellido et al. in an experimental model of osteoarthritis in rabbits [19]. Comparable changes can also be found in humans in the early stages of progressive cartilage degeneration. In a cohort study of women aged 45–64 years, a significant increase in bone resorption markers was observed in individuals with progressive osteoarthritis [20]. High levels of bone resorption markers have also been found in middle-aged individuals (aged 27–56 years) in the early stages of osteoarthritis without clinically manifested disease symptoms [21]. The causes of increased remodeling in the early stages of osteoarthritis are not fully understood but are believed to involve humoral interaction between damaged articular cartilage and subchondral bone tissue mediated by vascular invasion and communication channels between cartilage and bone.

Increased levels of transforming growth factor β (TGF-β), insulin-like growth factor (IGF), interleukins 1 and 6 (IL-1 and IL-6), and prostaglandin E2 (PGE2) have been found in altered articular cartilage. These factors are involved in the humoral regulation of bone remodeling. An experiment on osteoblast cell cultures showed that osteoblasts in osteoarthritic knee joints produce six times more IL-6 and PGE2 than osteoblasts in healthy joints [22,23]. It has been confirmed that changes in mineralization and volume of subchondral bone tissue occur below areas of articular cartilage that exhibit significant damage [24]. Microscopic lesions in subchondral bone tissue, even in healthy joints, can stimulate osteocytes to increase the production of receptor activator of nuclear factor-κB ligand (RANKL) and decrease osteoprotegerin (OPG), leading to increased bone resorption [25,26]. Decreased RANKL/OPG ratio and increased bone remodeling have been found in animals with induced OA development [19].

Remodeling of the subchondral bone tissue is accompanied by vascular invasion into the area of calcified cartilage. Increased vascularity is the result of the stimulation of blood vessels in the subchondral bone tissue by angiogenic factors, such as endothelial growth factor. Its concentration is significantly increased in the synovial fluid of OA patients [27]. Angiogenic factors stimulate chondrocytes in articular cartilage to synthesize and secrete matrix metalloproteinases and catabolic enzymes that prevent the interaction of metalloproteinases with their inhibitors. The combination of vascular invasion into the articular cartilage and increased influx of catabolic factors without inhibition of metalloproteinases ensures the progression of cartilage destruction. Consequently, these events diminish the mechanical integrity of the articular cartilage and promote remodeling as an attempt at joint adaptation to increased load. The existence of physiological communication between subchondral bone tissue and articular cartilage has been demonstrated in rats [28]. An experimental model of osteoarthritis in mice confirmed an increase in the caliber and number of these communications [29,30].

In the later stages of osteoarthritis, the intensity of remodeling decreases. Morphological features of such remodeling include increased bone volume and increased bone density, known as bone sclerosis. Both changes are clearly visible on X-ray images. By comparing the bone volume and density of OA patients with those of healthy individuals, it has been determined that OA patients have a 15% increase in bone density and a 30% increase in bone volume [31,32,33]. Analysis of subchondral bone tissue in OA patients has shown increased volume and reduced mineralization [34]. The inverse relationships of these bone tissue parameters can be interpreted as an adaptive attempt to compensate for decreased mineralization by increasing the volume of bone tissue [24]. Reduced mineralization of bone tissue during OA is associated with the structure of collagen produced by osteoblasts in osteoarthritic joints. It involves collagen type I composed of α1 chains, which distinguishes it from normal collagen type I composed of two α1 chains and one α2 chain [35,36]. Such collagen structure contributes to reduced mineralization during OA. Reduced bone resorption in the later stages of osteoarthritis is not accompanied by a decrease in osteoproduction, resulting in an overall increase in the volume and density of the subchondral bone tissue.

The described changes have secondary effects on surrounding joint tissues, particularly on the synovial membrane. In the later stages of OA, synovial membrane hyperplasia can occur without an inflammatory process.

## 3. Osteophytes Morphology and Development

Roland and Moskowitz consider osteoarthritis (OA) as the final stage of heterogeneous etiopathogenetic events affecting the joints [37]. In joints affected by OA changes, in addition to disturbances in the structure of the articular cartilage and subchondral bone tissue, osteophytes develop [38]. Osteophytes are bony outgrowths covered with a fibrocartilaginous cap (from Greek “osteo” = bone + “phyton” = plant, vegetation). Genuine osteophytes (osteochondrophytes) grow under the periosteum, along the edge of the articular cartilage or the insertion of the synovial membrane. In this regard, osteochondrophytes are called marginal osteophytes. The position of marginal osteophytes corresponds to the area where three different joint structures come into contact (articular cartilage, synovial membrane, and periosteum). The synovial fluid enables an additional indirect relationship between these tissues. Marginal osteophytes are a common finding in degenerative OA.

In addition to genuine osteophytes, traction and inflammatory osteophytes have been described. Traction osteophytes develop in the areas of tendon insertions (enthesophytes), while inflammatory osteophytes (syndesmophytes) are characteristic of ankylosing spondylitis. Osteophytes that appear further from the edge of the articular cartilage are called central osteophytes. Their position corresponds to the areas of damaged articular cartilage.

Osteophytes are clearly visible on X-ray images of joints affected by OA. Their distribution and dimensions can be precisely analyzed on appropriate joint radiographs.

The cause that triggers the development of osteophytes has not been established. The development of osteophytes involves morphogenetic processes from the early stages of skeletal development (endochondral and intramembranous ossification). Cytomorphologically and according to gene expression patterns, chondrogenesis and the formation of new bone tissue during osteophyte development particularly resemble identical processes in fracture healing with callus formation [39]. Possible causes of osteophyte development include mechanical overload of the joint and humoral factors. Experimental models of OA are based on these factors, such as cruciate ligament transection in the knee joint (biomechanical) and the administration of TGF-β1 and bone morphogenetic protein 2 (BMP-2) (humoral) into the joint cavity [40,41]. Destruction of the articular cartilage and the development of osteophytes have also been found in biomechanically unloaded joints during immobilization [42]. Therefore, previous notions that osteoarthritis is a causal consequence of aging have been rejected because cartilage atrophy alone does not always result in osteoarthritis. BMPs are a subset of the TGF-β superfamily of signaling molecules, and play a crucial role in regulating various cellular processes, particularly in the development and maintenance of bones, cartilage, and other tissues. BMP-2 promotes bone formation by stimulating the differentiation of mesenchymal stem cells into osteoblasts, facilitating the creation of new bone tissue [43,44].

Since the cause of osteophyte development has not been determined, despite the existence of experimental models, the question arises whether it is a functional adaptation of the joint or a pathological change. The answer still relies on assumptions. One theory suggests that osteophytes are the organism’s attempt to stabilize a biomechanically insufficient joint by increasing the surface area of the articular cartilage [45]. As OA progresses and articular cartilage degenerates, it is believed that osteophytes represent an adaptive attempt by the organism to repair the degenerated cartilage rather than a degenerative change [46]. It is probable that the local humoral milieu, established through certain biomechanical stimuli, stimulates chondrogenesis and endochondral ossification, leading to osteophyte formation [45].

In animal experimental models of OA, stages of osteophyte development have been described. The dominant mechanism of osteophyte formation is endochondral ossification, in which osteogenic cells mainly originate from the periosteum. Areas of intramembranous ossification appear during the definitive shaping of osteophytes, where osteogenic cells mainly originate from the synovial membrane. The expression of numerous growth factors has been investigated in osteophytes [45]. Research results regarding the expression of bone morphogenetic proteins in osteophytes are ambiguous. In rat periosteal stem cells, TGF-β did not induce chondrogenesis, while treatment of these cells with BMP-2 resulted in the production of collagen type II and aggrecan. Chondrocytes treated with BMP-2 showed markers of chondrocyte hypertrophy (collagen type X, osteocalcin), while in the culture treated with a combination of BMP-2 and TGF-β, chondrocyte hypertrophy was absent [47]. However, Uuistalo et al. showed that BMP-2 stimulates chondrogenesis from mouse periosteal stem cells and supports further endochondral ossification [48]. In a study on avian species, BMP-2 did not exhibit a chondrogenic effect on periosteal cells [49]. Chondrogenic differentiation of human mesenchymal stem cells from the synovial membrane was observed after the addition of TGF-β to the culture. Joyce et al., studying periosteal ossification in rats, found that depending on the dosage, TGF-β directs ossification in an endochondral or intramembranous direction. High doses of exogenous TGF-β introduced into the periosteum support endochondral ossification, while at lower doses, ossification mainly occurs intramembranously [50,51].

## 4. Radiological Characteristics of Osteoarthritis

It is believed that changes in subchondral bone tissue play a crucial role in the pathogenesis of osteoarthritis and can be detected early, long before clinical signs appear [52,53]. In the early stages of osteoarthritis, radiological signs are sparse, so depending on the stage of osteoarthritis, it is necessary to choose the appropriate radiological examination method. The expression of the radiological signs depends on the biomechanical characteristics of the affected joint.

**Figure 1:**
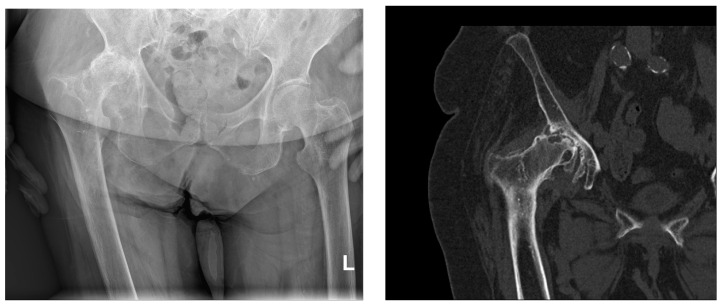
Sagittal X-ray of the pelvis and computerized tomography of the right hip (same patient). Severe right-sided coxarthrosis deformans. Grossly reduced joint space. Dense, sclerotic subchondral bone tissue of the deformed articular bodies interspersed with cyst-like transparencies of bone. Abundant marginal osteophytes of the articular surfaces and femoral head subluxation with consequent higher position of the right half of the pelvis. L—left side.

As degenerative changes progress, the articular cartilage deteriorates, and the joint space decreases, which is evident on X-rays as a narrowing of the joint space. The joint surfaces become deformed, flattened, uneven, or irregularly shaped. The latter is the result of focal cartilage proliferation that calcifies, leading to double or triple contour deformities on X-rays (Figure 1). Subchondral sclerosis of the joint surfaces can progress to eburnation (ivory-like appearance). Convex joint bodies lose their convexity, while concave joint bodies become shallower and flattened. Hypertrophic changes in the form of osteophytes develop chronically along the edges of the joint surfaces. Increasing osteophytes can result in a bony bridging between the articulating joint surfaces. Bone pseudocysts appear as cyst-like, marginally sclerotic transparencies in the subchondral area, varying in size and shape. Enlarged pseudocysts can cause subluxation of the joint bodies and the presence of loose bone fragments, called joint mice, in the joint space. Fracture of the bony wall of a pseudocyst can create communication between the joint cavity and the denuded joint body. Since 1957, the Kellgren and Lawrence classification has been used for the assessment of osteoarthritis changes in radiological practice. The radiological analysis includes osteophytes, periarticular ossifications, changes in articular cartilage associated with subchondral sclerosis of bone tissue, and pseudocysts in the subchondral bone tissue. According to the radiological findings, osteoarthritis changes are graded into five stages (0–4) [54].

Complete joint ankylosis as a result of osteoarthritis is rarely seen today. Cartilaginous elements in some joints often become calcified (chondrocalcinosis which should be differentiated from calcium pyrophosphate dihydrate deposition disease), such as the menisci in the knee joint or the glenoid labrum in the shoulder joint. The radiographic findings often do not correspond to the subjective complaints of the patients, so in the presence of a rich physical examination and clinical picture, the radiographic findings may be only minimally altered. Computed tomography, compared to conventional radiographic imaging of joints affected by osteoarthritis, provides richer data on the extent of the pathoanatomical substrate.

**Figure 2:**
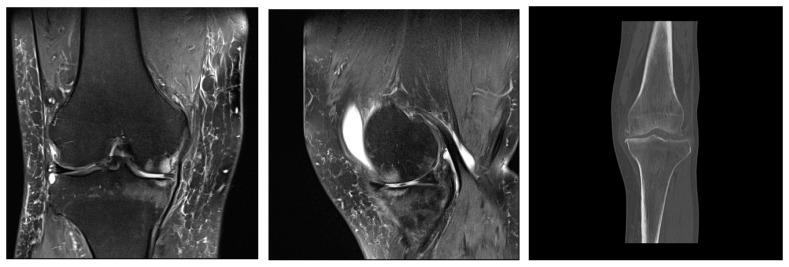
Mild deforming gonarthrosis visualized by MRI (proton-density fat saturated sequences in sagittal and coronal plane) and by CT (multiplanar reconstruction, coronal plane). Patchy areas of subchondral bone edema of femoral and tibial articulations with numerous marginal osteophytes. Articular cartilage is denuded in medial compartment, while in lateral compartment I-II degree hondromalacic changes are seen. Narrowed articular spaces with reactive effusion propagating to suprapatellar bursa are seen. High signal intensity of menisci and cruciate ligaments suggestive of degenerative changes. Ruptured medial meniscus. Reactive edema of periarticular and subcutaneous soft tissues.

In early osteoarthritis changes, visible on magnetic resonance imaging (MRI), there are subchondral sclerosis and cysts in the bone tissue, lesions in the surrounding bone marrow, fissures in the articular cartilage (Figure 2), and changes in the synovial membrane [55]. 

**Figure 3:**
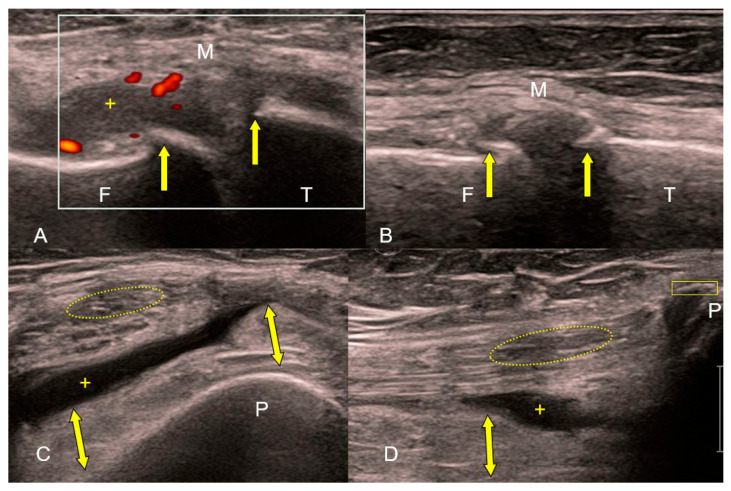
(**A**–**D**) Ultrasound of the knee joint in exacerbation of osteoarthritis (high-frequency linear probe: B-mode and Power Doppler). Irregular echoes from the femoral (F) and tibial (T) articular surfaces corresponding to marginal osteophytes (**A**,**B**). Hyperemia (red color in white rectangle) and edema of the thickened, hypertrophic synovial membrane (yellow bidirectional arrows) with effusion (+) in the suprapatellar bursa are signs of reactive inflammation. Loss of tension and fine linear echostructure of the medial collateral ligament (M). Absence of linear echostructure and decreased echogenicity (yellow ellipse) in the distal part of the quadriceps muscle tendon. Enthesophyte (yellow rectangle) at the base of the patella (P) (**C**,**D**).

**Figure 4:**
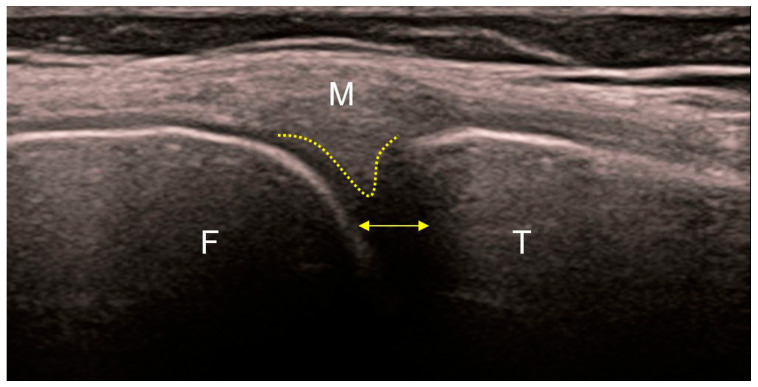
Ultrasound of the medial part of a healthy knee. Regular echoes from the femoral (F) and tibial (T) articular surfaces. The peripheral part of the medial meniscus (yellow dotted line) without a visible boundary continues into the posterior (oblique) fibers of the medial collateral ligament (M) that insert into it. The other (anterior) fibers of the medial collateral ligament continue vertically toward their insertion on the proximal tibia. Normal distance between the articular bodies indicates preserved thickness of the articular cartilages (yellow bidirectional line).

**Figure 5:**
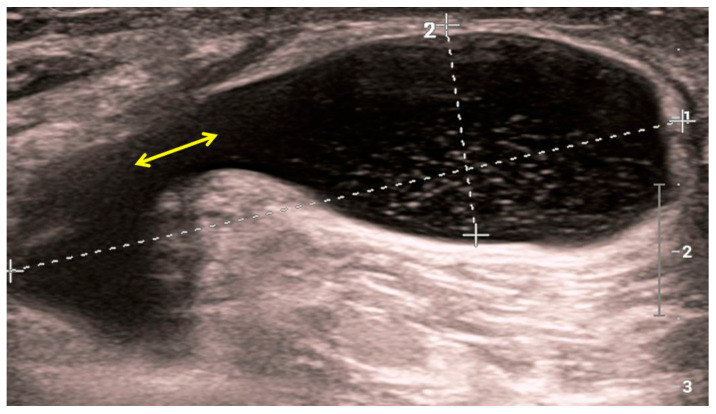
Sonographic image of a Baker’s cyst in the medial part of the popliteal fossa. Punctiform internal echoes from the cyst lumen which communicate with the joint space of the knee joint (yellow bidirectional arrow). Fluid within the cyst enhances the ultrasound beam posteriorly.

The role of ultrasound examination of the joints primarily relates to non-skeletal tissues (periosteum, synovial membrane, tendons, ligaments, etc.). Ultrasound can differentiate marginal bone appositions on joint bodies (osteophytes) and the width of the joint space, as well as its expansion due to effusion (Figure 3). Some components of the accessory structure of the joint, such as the meniscus in the knee joint, can be partially visualized with ultrasound (Figure 4). Synovial bursae adjacent to joints affected by osteoarthritis serve as an anatomical substrate for the development of cysts that can be well examined by ultrasound. Examples include the bursae in the popliteal fossa (bursae mucosae regionis genus posterioris), among which the bursa musculi semimembranosi and the bursa subtendinea musculi gastrocnemii medialis, often combined into one, from which a Baker’s cyst develops in 10–20% of cases of knee osteoarthritis. When a Baker’s cyst communicates with the joint space of the knee joint, inflammatory processes from the cyst can directly transfer to the knee joint (Figure 5). 

Numerous national and international guidelines have been developed for the diagnosis of osteoarthritis, which has been well-received by primary healthcare physicians. According to the British national guidelines from 2022, the diagnosis of osteoarthritis is made in individuals over 45 years of age with joint pain associated with physical activity, if they do not have morning stiffness of the joints or if it does not last longer than 30 min. The routine use of imaging diagnostic methods is not recommended, except in atypical cases that suggest a different diagnosis. This recommendation is supported by the lack of evidence for the contribution of imaging methods in diagnosing osteoarthritis, considering that anamnesis and physical examination of the patient are sufficient for diagnosis, while reducing unnecessary use of healthcare and financial resources [56]. A somewhat different point of view is held by a group of international experts, who believe that a positive clinical and typical radiographic finding is sufficient for diagnosing knee osteoarthritis, and further diagnostic imaging with magnetic resonance imaging is not recommended [57].

## 5. Conclusions

Contemporary experimental and clinical studies on osteoarthritis (OA) provide findings that are practically applicable to the pharmacological curative and rehabilitative approach to patients. Experts in this field unanimously emphasize the importance of a multidisciplinary and multimodal approach to OA casuistry, as well as an individualized approach to the patient. Interdisciplinary collaboration places significant demands on clinical radiology. It is expected to provide radiological diagnosis and data on the characteristics of the pathoanatomical substrate not only of the skeletal system but also of other affected locomotor apparatus systems. Radiology also includes the classification of OA changes, which, in addition to its diagnostic significance, serves as a starting point for deciding on a curative approach. The quantification of the effects of treatment and rehabilitation is also within the domain of radiological examination.

In recent times, human life expectancy has been increasing, as well as the duration of degenerative changes, which amplifies and complicates their consequences, affecting not only the health but also the economic status of patients and the entire community. For this reason, research in the field under discussion gains a broader foundation and deeper significance.

## Data Availability

Data used for analysis are contained within the article.
